# Key results from the first plasma operation phase and outlook for future performance in Wendelstein 7-X

**DOI:** 10.1063/1.4983629

**Published:** 2017-05-18

**Authors:** Thomas Sunn Pedersen, Andreas Dinklage, Yuriy Turkin, Robert Wolf, Sergey Bozhenkov, Joachim Geiger, Golo Fuchert, Hans-Stephan Bosch, Kian Rahbarnia, Henning Thomsen, Ulrich Neuner, Thomas Klinger, Andreas Langenberg, Humberto Trimiño Mora, Petra Kornejew, Jens Knauer, Matthias Hirsch, the W7-X Team, Novimir Pablant

**Affiliations:** 1 Max Planck Institute for Plasma Physics, Greifswald, Germany; 2 Princeton Plasma Physics Laboratory, Princeton, New Jersey 08543, USA

## Abstract

The first physics operation phase on the stellarator experiment Wendelstein 7-X was successfully completed in March 2016 after about 10 weeks of operation. Experiments in this phase were conducted with five graphite limiters as the primary plasma-facing components. Overall, the results were beyond the expectations published shortly before the start of operation [Sunn Pedersen *et al.*, Nucl. Fusion **55**, 126001 (2015)] both with respect to parameters reached and with respect to physics themes addressed. We report here on some of the most important plasma experiments that were conducted. The importance of electric fields on global confinement will be discussed, and the obtained results will be compared and contrasted with results from other devices, quantified in terms of the fusion triple product. Expected values for the triple product in future operation phases will also be described and put into a broader fusion perspective.

## INTRODUCTION

**FIG. 1.**
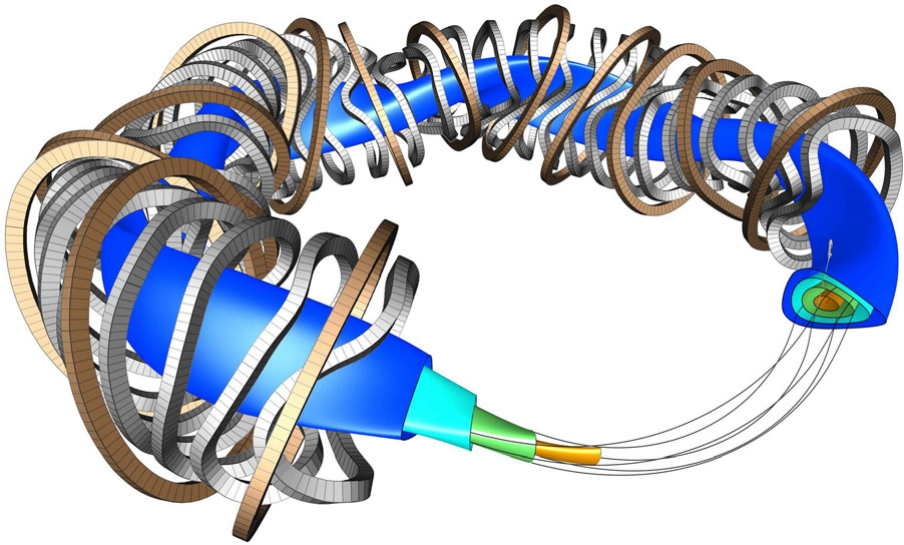
Representative flux surfaces, field lines, and part of the superconducting coil set of W7-X are shown in this CAD drawing.

The Wendelstein 7-X (W7-X) experiment^1^ is the most advanced stellarator in the world today. With confinement volumes of approximately 30 m^3^, the Large Helical Device (LHD) heliotron and W7-X stellarator share the status of being the largest stellarators taken into operation to date (we use the word stellarator in this article to refer to both heliotrons and stellarators). W7-X aims to show the fusion-reactor relevance of optimized stellarators, in particular, that the intrinsic benefits of the stellarator can be combined with reactor-relevant, tokamak-like confinement, also at plasma parameters close to those of a reactor, $T_{e0}\geq T_{i0}>4$ keV, $n_{e0}>10^{20}$ m^–3^, ⟨β⟩≥5%. Here, *β* denotes the normalized total plasma pressure $2\mu_{0}(p_{e}+p_{i})/B^{2}$ and “⟨⟩” the volume average. Intrinsic advantages include steady-state operation, lack of major disruptions, no risk of significant runaway electron generation, and no need for current drive.^1^ The device, which is five-fold symmetric, features 70 superconducting NbTi magnets, two planar coils, and five non-planar coils in each of the 10 half-modules. The coil system is designed to allow operation up to $B_{0}=3.0$ T on axis, but is operated at $B_{0}=2.5$ T, the resonant field for second-harmonic absorption (X2 and O2) of the ECRH system that operates at *f* = 140 GHz. The fundamental layout is shown in Figure 1.

The magnetic surfaces have a major radius *R* = 5.5 m and an average minor radius of *a* = 0.5 m. The peculiar shapes of the non-planar coils are the result of solving the Biot-Savart law $\nabla \times \vec{B}=\mu_{0}\vec{j}$ to create discrete, modular coils that generate the required 3D magnetic vector field coming out of the physics optimization described in “Stellarator Optimization and Recent Achievements in the World Stellarator Program” section.^2,3^ The planar coils are used to add or subtract toroidal magnetic field components to lower or increase the rotational transform ι=1/q, where *q* is the *safety factor* known from tokamak physics. In a stellarator, the magnetic field is created dominantly by the external coils, including the poloidal component that gives rise to *ι*, the twist of the magnetic field lines on a magnetic surface. In a tokamak, *ι* results from a combination of poloidal field from the toroidal plasma current and toroidal field from the poloidal currents in the external toroidal field coils. The planar coils can also be used to increase or decrease the toroidal magnetic strength variation, so as to change the mirror ratio in the device.

## STELLARATOR OPTIMIZATION AND RECENT ACHIEVEMENTS IN THE WORLD STELLARATOR PROGRAM

The stellarator concept has intrinsic advantages as a fusion power plant concept. The lack of a strong toroidal current means that no current drive is necessary, that the concept is intrinsically steady-state-capable, and that there are no major problems with disruptions or run-away electrons. However, concerns remain about stellarator confinement at high ion temperatures, and fewer stellarators than tokamaks have been built, and none of them as large as the largest existing tokamaks. For example, JET has a confinement volume of about 100 m^3^, over a factor of three more than W7-X and LHD.

It has been known for more than 30 years that stellarators can suffer from unconfined drift orbits of the magnetically trapped particles. With increasing temperature, neoclassical transport can dominate over turbulent transport because $\chi \propto T^{3.5}$, with *χ* being the heat transport coefficient. This seemed to preclude the simultaneous achievement of high *T_i_* and high *τ_E_* (the energy confinement time) and therefore the reactor prospects of stellarators, if these drift orbit losses could not be significantly reduced. In the early 1980s, new ideas emerged how to improve the drift orbit confinement by tailoring the magnetic field. At that time, it was also realized that one could also reduce intrinsic equilibrium currents parallel to the magnetic field, such as Pfirsch-Schlüter and bootstrap currents. This progress in theory, together with advances in the supercomputing capabilities and numerical algorithms, allowed identification and optimization of specifically tailored stellarator topologies that have vastly improved drift orbit confinement. These new configurations are referred to as optimized stellarators, whereas the non-optimized stellarators are referred to as classical stellarators. Several different optimization approaches exist. The reader is referred to two recent reviews for more information on stellarator optimization strategies and their theoretical foundations.^4,5^

The stellarator Wendelstein 7-AS, operated from 1988, was a first test of the optimization effort but realized only some of the optimization ideas and is therefore often referred to as partially optimized. Nevertheless, it delivered many encouraging results before it was shut down in 2002,^6^ results that supported the idea that stellarator optimization can be effective.

An important discovery for stellarator optimization is quasi-symmetry: When viewed in Boozer coordinates,^7^ the particle orbits depend only on the magnitude of B, which can be tailored to have a symmetry—a direction along which the magnetic field strength does not change. The symmetry cannot be achieved perfectly on all flux surfaces, so it is called quasi-symmetry. The first quasi-symmetric experiment was the Helically Symmetrix eXperiment HSX, which started operation in 1999.^8^ As the name implies, its direction of quasi-symmetry is helical (as opposed to, e.g., toroidal). HSX has successfully demonstrated several of the key predictions such as the reduced damping of plasma flow in the quasihelical direction^9^ and improved neoclassical confinement.^10^ The combination of its small minor radius (a = 12 cm) and its relatively low plasma densities ($n_{e}<10^{19}$ m^–3^) prevents it from proving the optimization in terms of a high energy confinement time simultaneously with a high ion temperature, since its plasmas were heated by ECRH and its densities are so low that only about 5% of the heating power is transferred to the ions, and charge exchange losses are a significant sink for the ion thermal energy. LHD is a superconducting 3D configuration of the torsatron/heliotron type that went into operation in 1998. It displays some features of optimization, in particular, in its so-called *inward shifted* configuration.^11^ It is large enough to not be affected significantly by charge exchange losses, can operate at high densities and with direct ion heating, and has achieved high performance, including $\langle \beta \rangle =5.1\%,\;T_{i}=8.1$ keV, $T_{e}=$ 20 keV, $n_{e}=1.2\times 10^{21}$ m^–3^, and steady state operation (pulse times exceeding 1 h). The device has not achieved these parameters simultaneously. For example, the impressive electron density, possible because a stellarator has no Greenwald density limit, was achieved at very modest temperatures T≈0.25 keV. W7-X does not aim particularly to break any of these impressive records, but rather to show the *simultaneous* achievement of $T_{e}\geq T_{i}\geq 4$ keV, ⟨β⟩=5%, and $n_{e0}=2\times 10^{20}$ m^–3^. Such simultaneous values appear achievable based on the transport simulations, which we will present later in this paper.

## RESTRICTIONS IN FIRST OPERATION PHASE (OP1.1) DUE TO LIMITER OPERATION

The operation phase 1.1 (OP1.1) was first and foremost an integral commissioning of the entire device, including diagnostics and heating systems. The installation of plasma-facing components (PFCs) was held to a minimum, with only five symmetrically placed inboard graphite limiters, so that first physics results could be gained quickly, and any poorly performing components could be identified early and, if needed, be upgraded, during the installation period preceding the next operation phase, OP1.2. OP1.2 will feature a full set of (un-cooled) divertors. The limiters and the expected physics program for OP1.1 are described in an article that was published shortly before first plasma.^12^ The design of the limiters is described in more detail in an upcoming article.^13^ To note here is that the use of uncooled graphite limiters was expected to limit the pulse length to 2 MJ, and that the full density control was not expected since the limiters did not provide the efficient particle exhaust capabilities that is expected in the future divertor operation.^14,15^ Indeed, almost all the physics topics presented in that paper were addressed successfully, and it was possible to extend beyond them as well.

## SUCCESSIVE IMPROVEMENTS IN PLASMA QUALITY AND DURATION IN OP1.1

For all discharges in the first weeks of operation, the edge plasma radiated the heat away so effectively that the plasma barely had contact with the limiters. This was confirmed with the video diagnostic, infrared cameras, Langmuir probes, and thermocouples. Plasmas would expand in radius initially but suffer a slow radiation collapse before having expanded to the last closed flux surface, due to the release of water vapor and other wall impurities.

Wall conditioning was improved over time simply by creating discharge after discharge of helium plasma, each one releasing wall impurities and the next discharges living progressively longer as the walls slowly cleaned up. Once fully operational, glow-discharge cleaning was also used and provided a more efficient wall-conditioning.^16^ Discharges began to extend to the limiters and had prolonged contact with them, as the pulse lengths continued to increase. As the working gas was switched from helium to hydrogen about midway through the run campaign, the plasma parameters and behavior continued to improve rather continuously as the walls progressively cleaned up. Curiously, wall conditions would deteriorate during a run day, and neutral pressure spikes would terminate the plasma through a radiation collapse earlier and earlier in the discharge. The explanation for this behavior is still under investigation but is consistent with there being some reservoir of primarily hydrogen, which progressively warms up from discharge to discharge and eventually reaches a temperature where it begins to evaporate, since the neutral gas release occurred at an earlier point in the discharge than the previous discharge. The good performance could be partly recovered with one or two helium discharges and fully recovered on the following run day after glow discharge cleaning. Even for the first discharges of a run day, which had prolonged contact with the limiters, at most 60% of the heating power ended up on the limiters, the rest was deposited elsewhere through some combination of radiation and charge-exchange neutral losses. A quantitative assessment of these other loss channels is left to future publications.

An important consequence of this was that the pulse limit of 2 MJ turned out to be overly conservative—there was no evidence of limiters being anywhere near their limits. Therefore, it was agreed to increase it to 4 MJ for the last two weeks of operation. This allowed for longer pulses, in particular, for ones that lasted as long as 6 s, some of which will be highlighted in the following.

## THE TWO MAGNETIC CONFIGURATIONS IN OP1.1

**FIG. 2.**
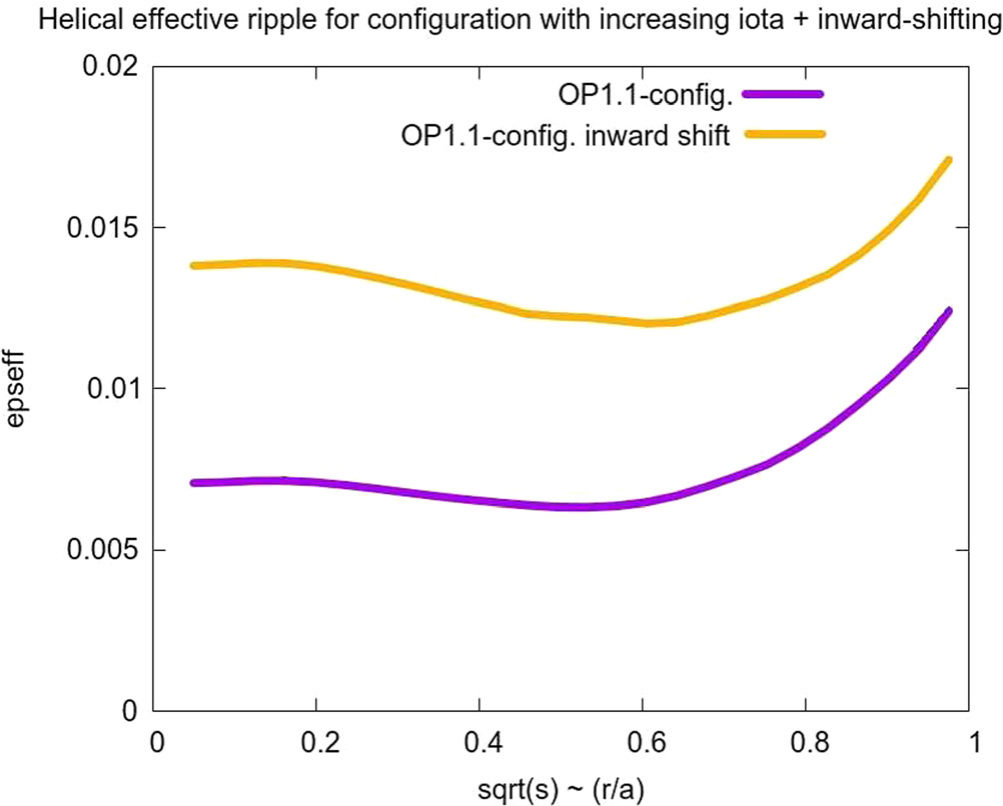
Profiles of $\epsilon_{\text{eff}}$ for the standard OP1.1 configuration and the alternative configuration.

As previously described,^12^ a special magnetic configuration was chosen in OP1.1 to ensure that >99% of the convective heat loads would end up on the five inboard limiters. This was done by adjusting the rotational transform ᵼ at the edge and the near-SOL to be far from the resonances ᵼ=5/5 and ᵼ=5/6, which are both associated with substantial island chains given the designed-in *n* = 5 toroidal component of the magnetic field. The vast majority of OP1.1 discharges were performed in this configuration. A second configuration was also used during the last run week of OP1.1, as well as several configurations in between these two. This alternative configuration had a slightly higher ᵼ value and significantly higher helical ripple $\epsilon_{\text{eff}}\approx 0.0137$ as compared to $\epsilon_{\text{eff}}\approx 0.0070$ (see Figure 2).

## ACHIEVED AND PREDICTED TRIPLE PRODUCTS FOR W7-X

The figure of merit for producing net power in a fusion reactor is the triple product $n_{i}T_{i}\tau_{E}$, which must exceed appr. $3\times 10^{21}$ m^–3^ keV s for a fully self-heated D-T fusion plasma.^17,18^ This is not the only requirement. Others include having *T_i_* roughly in the range of 10–40 keV, and, if the plasma is to be self-heated by the *α*-particles, having $T_{e}\geq T_{i}$, since the electrons receive the majority of the *α*-particle power. Although not an absolute must, the requirement of having sufficiently long pulses, or even steady-state operation, is highly desirable, and this is indeed one of the major advantages of the stellarator concept. Although these other requirements have to be kept in mind, the triple product is a highly relevant and useful scalar measure of progress. Within a tokamak context, apart from the triple product, focus has been on the simultaneously achieved central ion temperature $T_{i0}$. The achieved pulse length has been less of a focus, but pulse length extension and steady-state operation at high performance are active areas of research in tokamak physics today.

In order to evaluate how far the stellarators have progressed towards the ultimate goal of net power production, and also to provide an objective comparison to tokamak performance, we calculate in the following some achieved triple products in OP1.1, as well as some predicted triple products for future operation phases, and then compare them to existing and future expected achievements for tokamaks and stellarators in a plot of triple-product versus pulse length plot.

### Some achieved triple products, ion temperatures and pulse lengths in OP1.1

**FIG. 3.**
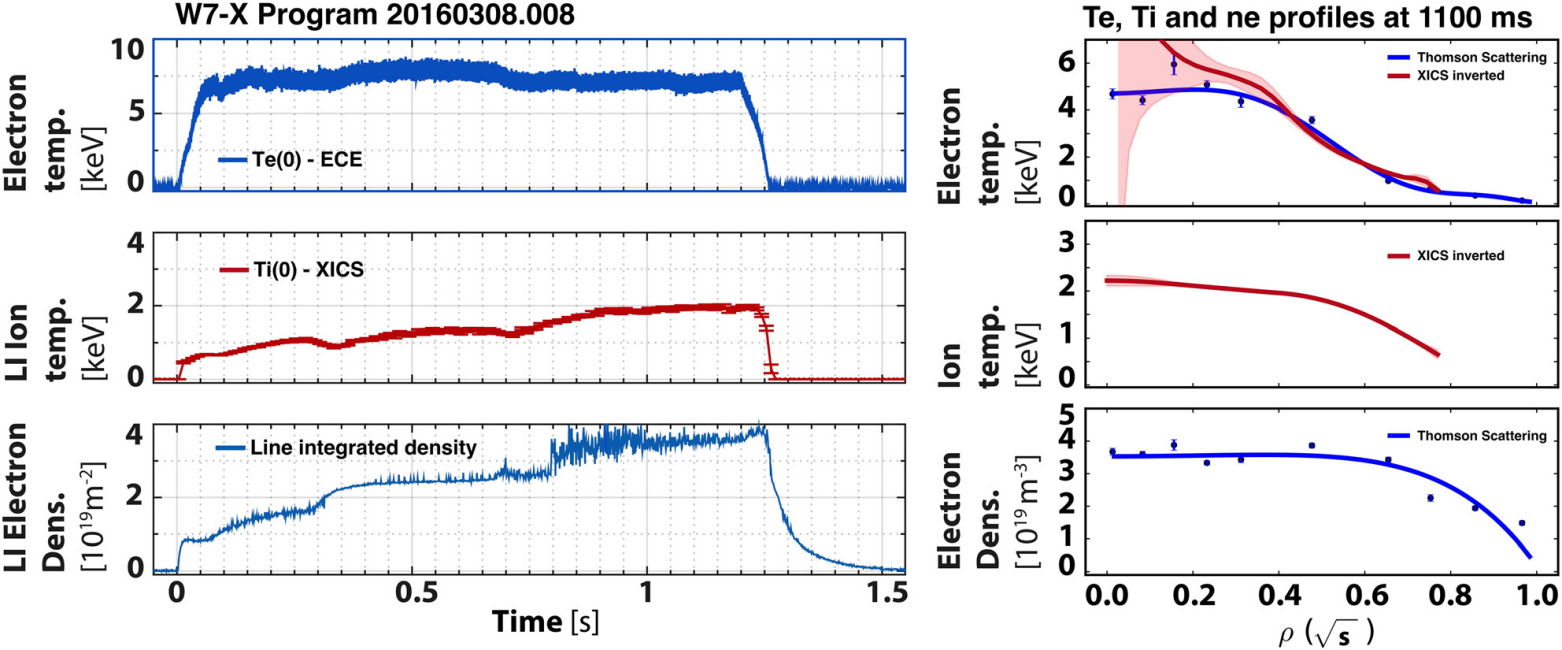
Time traces (left) and profiles (right) of electron and ion temperatures and electron density for a 1.3 s discharge, 20160308.008, heated continuously with 2.7 MW of ERCH. The profile data are taken at t = 1.1 s.

We focus here on three discharges, performed during the last three days of OP1.1 operation, where the wall conditions had improved enough that longer discharges with stable density and modest impurity radiation were achieved. The first discharge, 20160308.008, was a 1.3 s long discharge heated with 2.7 MW of 140 GHz ECRH X2 absorption (Fig. 3). At t = 1.1 s, the Thomson scattering (TS) system^19^ showed central electron density and temperature values of $n_{e0}=3.5\times 10^{19}$ m^–3^ and $T_{e0}\geq 4$ keV, respectively. The x-ray crystal imaging spectrometer (XICS) measured the central ion temperature to be $T_{i0}=2.2$ keV at that point in time. Using a combination of measurements from diamagnetic loops, TS, XICS, and other profile diagnostics, the energy confinement time is estimated at $\tau_{E}\approx 0.10$ s. At our present understanding of the consistency and calibration accuracy of the diagnostics, the absolute value of *τ_E_* is believed to be accurately 20%, whereas relative changes in *τ_E_* can be detected down to 5%–10%, in particular, with the diamagnetic loops. An overview of the various diagnostics in operation in OP1.1 is presented elsewhere.^20^ The triple product achieved was $0.8\times 10^{19}$ m^–3 ^keV s for this 1.3 s discharge. The electron temperature, although not part of the triple product, is given to stress the point that *T_e_* was substantially greater than *T_i_*.

**FIG. 4.**
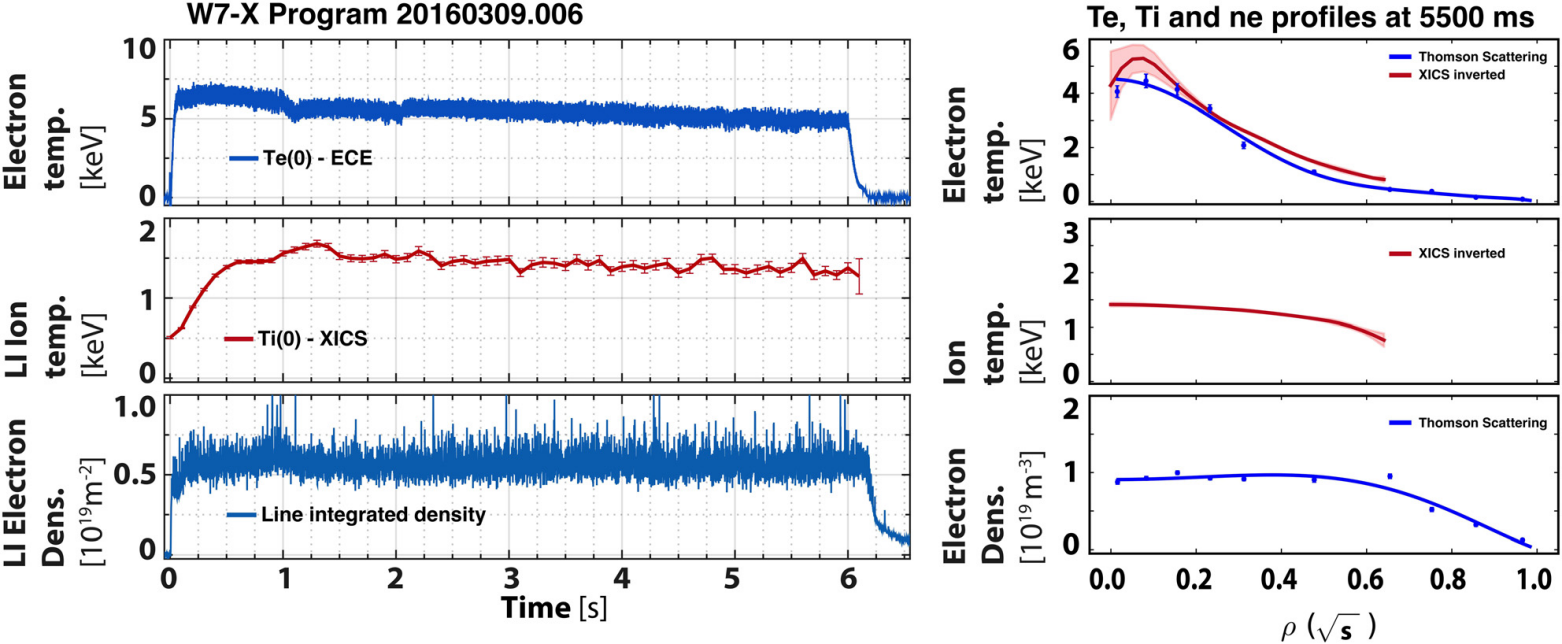
Time traces (left) and profiles (right) of electron and ion temperatures and electron density for a 6 s discharge, 20160309.006. The time trace for density is noisy due to suboptimal performance of the interferometer laser on that shot, not due to any unusually large density fluctuations. The profile data are taken at t = 5.5 s where the heating power was 0.6 MW.

**FIG. 5.**
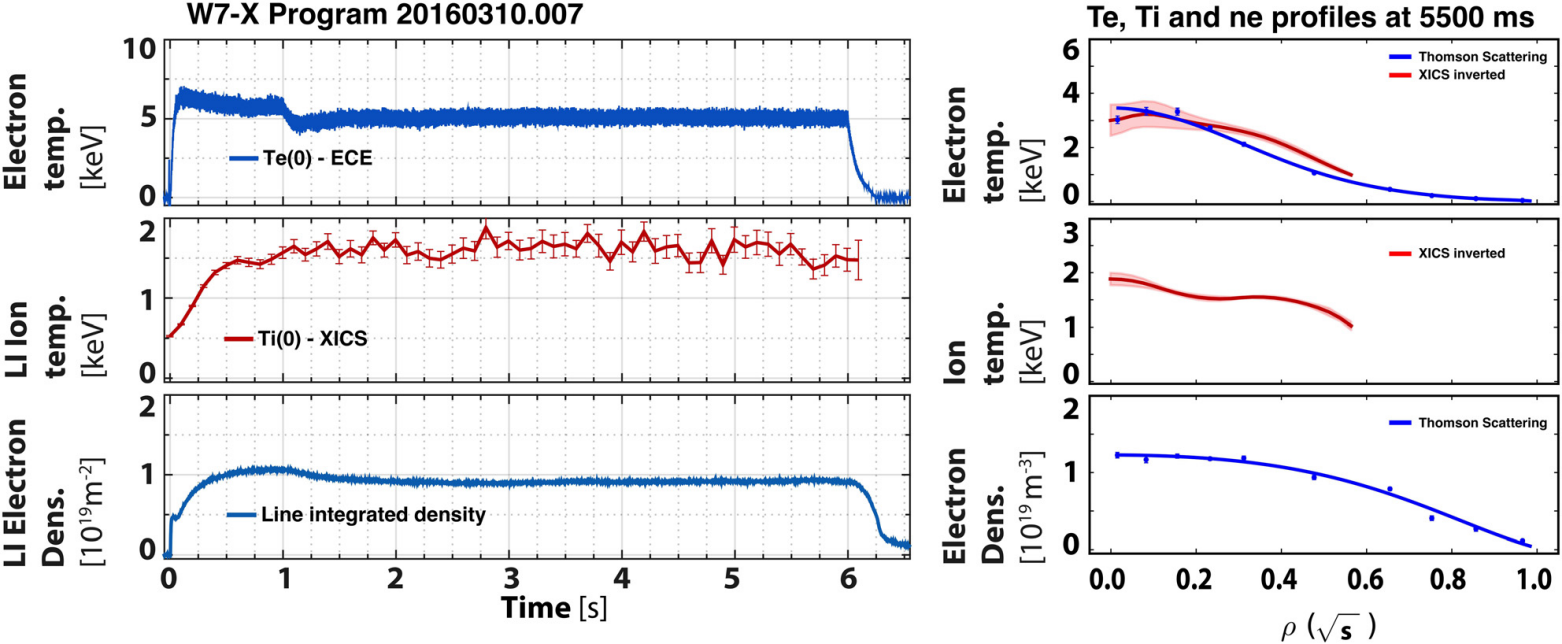
Time traces (left) and profiles (right) of electron and ion temperatures and electron density for a 6 s discharge, 20160310.007, in the alternative magnetic configuration. The profile data are also taken at t = 5.5 s where the heating power was 0.6 MW.

The second discharge, 20160309.006, Fig. 4, was a low-power 6 s discharge, which had a 1 s of $P_{\text{ECRH}}=1.1$ MW initial phase followed by 5 s of $P_{\text{ECRH}}=0.6$ MW. Towards the end of the lower-power phase, at t = 5.5 s, $n_{e0}=1.0\times 10^{19}$ m^–3^, $T_{i0}=1.4$ keV, $T_{e0}\approx 4$ keV, $\tau_{E}=0.125$ s, and a triple product of $0.18\times 10^{19}$ m^–3 ^keV s. Interestingly, the third discharge, 20160310.007, which had exactly the same heating power sequence, but used the alternative OP1.1 magnetic configuration with the higher $\epsilon_{\text{eff}}$, had the same or perhaps even slightly better confinement and a larger triple product. Data for this discharge are shown in Fig. 5. At t = 5.5 s, where $n_{e0}=1.3\times 10^{19}$ m^–3^, $T_{e0}\approx 3$ keV, $T_{i0}\geq 1.8$ keV, and $\tau_{E}=0.132$ s, a triple product of $0.27\times 10^{19}$ m^–3 ^keV s was reached.

We have assumed here, and also in the projections of triple products presented later in the article, that *n_i_* = *n_e_*, i.e., we have neglected the diluting effect of impurities. The analysis of impurity content in OP1.1 is ongoing, but preliminary results suggest that a few percent of low-Z impurities, in particular, carbon, was typically present, which would lead to a hydrogen ion density about 20% lower than the electron density and a corresponding 20% reduction in achieved triple products relative to those stated here. In future operation phases, higher plasma densities, the presence of a divertor, and a better conditioned first wall will presumably lead to lower impurity fraction.

The slightly higher *τ_E_* value (still within the uncertainty) is in contradiction to an $\epsilon_{\text{eff}}^{3/2}$ scaling, which would have the confinement time of the third discharge (20160310.007) by a factor of 2.7 smaller than the second discharge (20160309.006), and still a factor of more than 2 even if one takes into account that this discharge had a somewhat higher density. However, the result that *τ_E_* is essentially unaffected by $\epsilon_{\text{eff}}$ was in fact to be expected and will be explained in “Electric Fields and Their Effect on Particle Confinement in Toroidal Devices” section.

## ELECTRIC FIELDS AND THEIR EFFECT ON PARTICLE CONFINEMENT IN TOROIDAL DEVICES

The primary reason that the level of magnetic ripple does not affect transport in these plasmas is that the electric field, or more accurately the *E *×* B* drift, heals otherwise lossy guiding center drift orbits. It has been known for a while that radial electric fields can heal otherwise unconfined particle orbits in toroidal devices, e.g., in the pure toroidal field trap, as shown experimentally in the Lawrence Non-neutral Torus II (LNT-II).^21,22^ In stellarators, these healing effects have also been shown by studying non-neutral plasmas in the Columbia Non-neutral Torus (CNT), a classical stellarator.^23–25^ These effects were well known before LNT-II and CNT started operation. Already in 1983, Boozer *et al.* wrote:^26^ *It has been realized both computationally and, more recently, experimentally that the presence of an electric field is essential to good particle confinement in stellarators. E *×* B* effects are routinely included in neoclassical stellarator transport codes (see, e.g., Ref. 27). The basics of these effects are discussed in the following.

### When is *E* × *B* important?

One can assess the importance of the potentially healing effects of *E *×* B* on the drift orbits by comparing the magnitude of the magnetic drifts *v_B_* to the magnitude of the *E *×* B* drift, *v_E_* for a thermal particle at temperature *T* with charge q, assuming, for simplicity, that the gradient scale lengths for the electrostatic potential and the magnetic field strength perpendicular to B are similar

$$v_{E}/v_{B}=|\frac{\nabla \phi }{B}\frac{qB^{2}}{2T\nabla B}|\approx |\frac{q\phi }{2T}|.$$ (1)

For a pure electron plasma, this ratio is approximately equal to $L^{2}/\lambda_{D}^{2}$, with *L* being the gradient scale length of the electrostatic potential, as can be shown directly from Poisson's equation

$$\epsilon_{0}\nabla^{2}\phi =-en_{e}\Rightarrow \phi \approx -en_{e}L^{2}/\epsilon_{0}\Rightarrow \frac{\left|e\phi \right|}{T_{e}}\approx \frac{ne^{2}}{\epsilon_{0}T_{e}}L^{2}=L^{2}/\lambda_{D}^{2}.$$ (2)

*L* is typically of order the smallest dimension of the plasma (e.g., minor radius in a toroidal system), and therefore by the usual textbook small-Debye-length plasma definition, this ratio is much larger than one. Consequently, in a pure electron plasma, the E×B drift dominates strongly. In CNT pure electron plasmas, this ratio was about 25.^24^ For a quasineutral plasma, the ambipolarity constraint sets the size of |ϕ| and it is of order unity or smaller. In OP1.1, this ratio was relatively large, since *T_e_* exceeded *T_i_* substantially (e.g., in the 20160310.007 discharge mentioned earlier). Thus, the *E *×* B* drift had a substantial orbit-healing effect, in particular, for the ions, thus making the confinement insensitive to the factor of two increase in $\epsilon_{\text{eff}}$. As discussed in the next paragraph, for future operation phases, a smaller difference between electron and ion temperatures is expected, and therefore the electric field will be smaller, and the effects of the magnetic field optimization will be more pronounced. It is worth pointing out here that confinement of fusion *α* particles cannot be healed by the ambipolar electric field in a D-T fusion reactor plasma. Since the electrostatic potential is created by the bulk plasma, qϕ will be of order 20 keV, whereas the initial kinetic energy of the fusion *α* particles is 3.5 MeV. Thus, optimization of the magnetic field of a stellarator is certainly necessary for *α*-particle confinement.

The thermal plasma particle orbits are affected by the *E *×* B* drift, possibly significantly, given that the ratio in Eq. (1) is typically of order unity. The operating regime with $T_{e}\gg T_{i}$ and a strong positive radial electric field in the core are referred to as *core electron-root confinement* (CERC).^28^ As the word *core* implies, the electric field effects are important in the core. Near the edge, electric fields generally play less of a role. However, the lower temperatures and often stronger density gradients typically lead to a situation where the anomalous transport dominates over neoclassical transport, which also leads to insensitivity towards the value of $\epsilon_{\text{eff}}$. Thus, the global confinement becomes largely independent of $\epsilon_{\text{eff}}$.

The neoclassical transport is not necessarily subdominant even if the parts that scale with $\epsilon_{\text{eff}}$ are. Just as in a tokamak, a neoclassical transport remains even in the absence of transport driven by a magnetic ripple. At present, a detailed validated transport analysis is not available, and we cannot yet determine if the transport is dominated by neoclassical or anomalous transport processes, but it is worth noting that also for turbulent transport, electric fields play an important, sometimes decisive, role. It goes beyond the scope of this paper to go into any detail on this matter, but the reader is referred to the aforementioned paper on H-modes,^41^ as well as recent papers on zonal flows and the interaction between short- and long-range electric fields in stellarators.^29,30^

The W7-X optimization included a minimization of the bootstrap current, in order to make the edge island topology independent of plasma parameters, so that good island divertor operation will be possible for a range of plasma pressures. It is possible to optimize the neoclassical confinement simultaneously with a minimization of the bootstrap current,^31^ but a small bootstrap current remains in the configurations that are best optimized for neoclassical confinement—of order 50 kA at full performance (as compared to about 1 MA for a comparable tokamak plasma). Conversely, the bootstrap current can be brought to essentially zero with some degradation of the neoclassical confinement—the authors of Ref. 32 say, in reference to their study of already highly optimized configurations, “*For all the W7-X configurations under investigation, the minimisation of I_b_ is in conflict with the neoclassical confinement improvement.*”

**FIG. 6.**
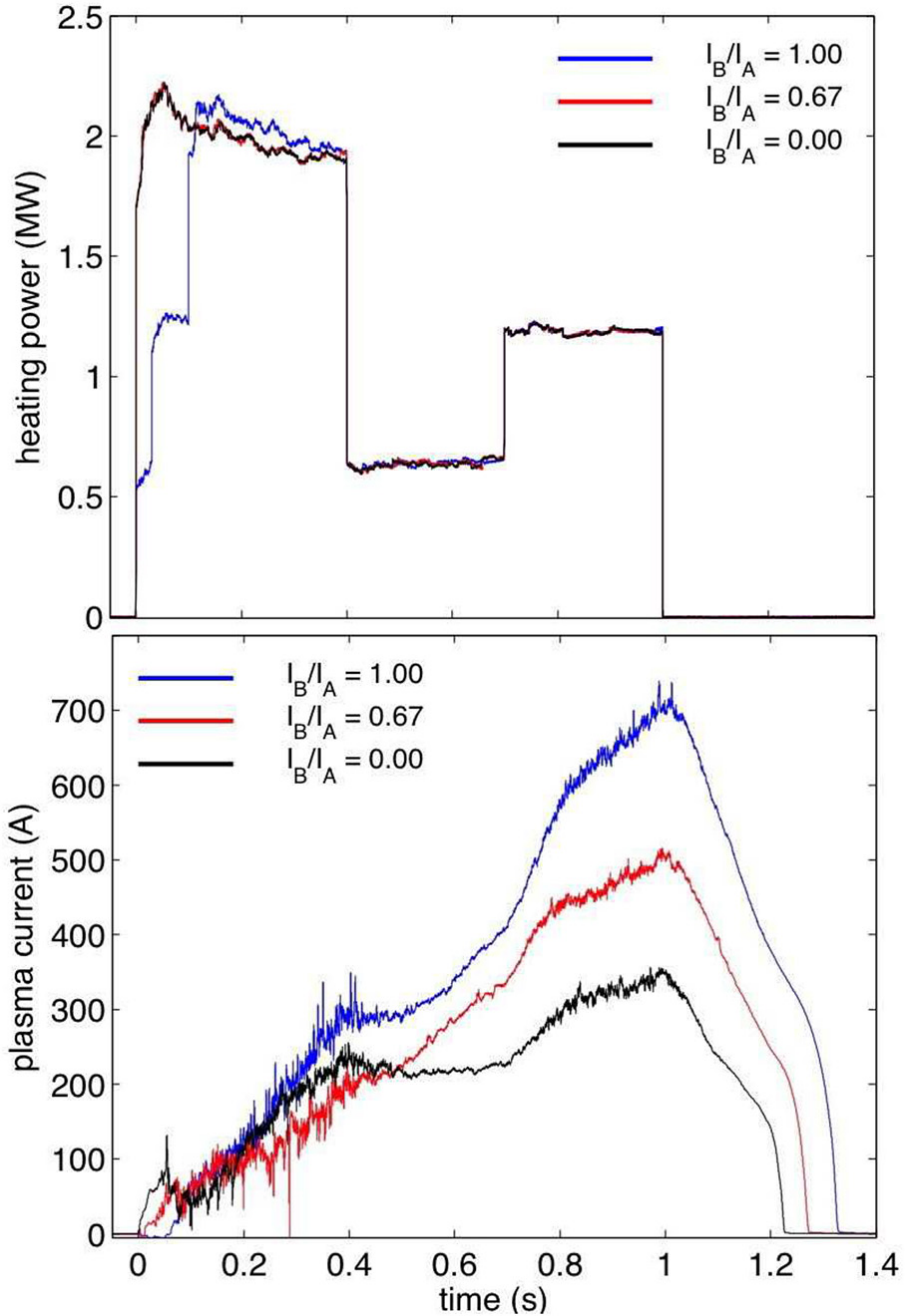
The nominally identical ECR heating programming (top) and the evolving bootstrap currents (bottom) for three different configurations, the standard OP1.1 configuration (blue) 20160309.010, the alternative OP1.1 configuration (black, 20160309.029), and an in-between configuration (red, 20160309.018). A clear tendency for lower bootstrap current in the alternative configuration is seen.

For the two configurations investigated in OP1.1, this negative correlation is also present. The alternative configuration, whose neoclassical ripple is significantly larger, is expected to have a lower bootstrap current. The bootstrap current remains sensitive to the magnetic configuration even in the presence of large electric fields, so one should be able to measure a larger toroidal current for this configuration. Indeed, in a scan of configurations with increasing mirror term, we see the expected clear and steady reduction of the toroidal current, as measured by Rogowski coils,^33^ as the current in planar coil type A is lowered successively, thereby increasing the mirror term and decreasing the expected bootstrap current (Figure 6). Three discharges are shown, all three having the same programmed power steps in ECRH, with some deviations in the first few hundred milliseconds in the actual injected power. The toroidal current is strongly evolving over time, since the bootstrap current depends on the kinetic profiles, which are also evolving, and additionally, the plasma generates opposing currents that decay over a characteristic L/R time which is on the order of several seconds (see, e.g., the numerical simulations in Ref. 12).

## EXPECTED TRIPLE PRODUCTS, ION TEMPERATURES AND PULSE LENGTHS IN OP1.2 AND OP2

In the following, we will evaluate the expected triple products for the future operation phases OP1.2 and OP2. OP1.2 will feature the test divertor units (TDU),^14^ ten un-cooled fine-grain graphite divertor units with the same geometric shape as the fully water-cooled carbon-fiber composite (CFC) divertor system foreseen for OP2, which will allow for steady-state operation with divertor surface heat fluxes up to 10 MW/m^2^.^15^ The main increases in the triple product will come from strong increases in *n_e_*, whereas the confinement times and ion temperatures are expected to increase, but only modestly beyond what was achieved in OP1.1, as explained in the following.

It has been found empirically, in stellarators as well as tokamaks, that increased density increases the confinement time. This can be seen in the stellarator (and tokamak) ISS04 scaling^34^ and the tokamak-only IPB98(y,2)^35^ scaling. For ISS04, $\tau_{E}\propto n^{0.54}$, IPB98(y,2): $\tau_{E}\propto n^{0.41}$, whereas increased heating power decreases the confinement time (ISS04: $\tau_{E}\propto P^{-0.61}$, IPB98(y,2): $\tau_{E}\propto P^{-0.69}$). Thus, expecting to increase heating power and density, confinement times will only change modestly, whereas the ion temperature is expected to go up substantially, since the increased density allows for better coupling between the electron and ion temperatures, and the increased heating, in particular, the use of direct ion heating (NBI and ICRH), also helps increase the ion temperature. NBI is planned to reach up to 7 MW in OP1.2 and 10 MW in OP2. Ion cyclotron resonance heating is planned at a rather modest level of 1–2 MW, thus playing a relatively minor role in the energy balance for the ions, but is expected to play an important role in the generation of fast ions (order 50 keV) on the inner magnetic surfaces, to verify their confinement. Experimental verification of good confinement of 50 keV deuterium ions is a major goal of W7-X since they are a good proxy for fusion *α* particle confinement in a stellarator reactor: For 50 keV deuterium ions at 2.5 T, the Larmor radius is $r_{L}\approx 1.3$ cm, i.e., $r_{L}/a\approx 0.026$, whereas the fusion *α* particles at 3.5 MeV in, e.g., the HELIAS 5-B reactor design^38^ at B = 5.5 T have $r_{L}\approx 3.5$ cm, i.e., $r_{L}/a\approx 0.019$, slightly lower. Therefore, the confinement of 50 keV deuterium ions in W7-X is a more than adequate proxy for *α*-particles in a stellarator fusion reactor.

### Operation at higher density

The plasma density is expected to be increased by a factor of about 4, as a result of a number of added device capabilities: having a better control of the neutral density at the edge by better wall conditioning, having a more efficient particle exhaust with a divertor, having more efficient core fueling using pellets, and having substantially more heating power. All these contribute to prevent radiative collapses or instabilities of various types that could prevent high-density operation.^36,37^ For a quantitative assessment of the future performance, we use a predictive one-dimensional code that calculates the neoclassical fluxes in the presence of a self-consistent electrical field, and an ad-hoc model for anomalous transport applied only to the edge region^39^ since it is assumed that anomalous transport will play a dominant role only in the edge region. The code takes a heat deposition profile, a density profile, and a $Z_{\text{eff}}$ profile as inputs and then calculates $T_{e}(r),\;T_{i}(r)$, *E*(*r*), and *τ_E_*. Due to its somewhat optimistic and not fully self-consistent assumption about anomalous transport, its results could be considered on the optimistic side of what should be expected. Results from HSX indicate that anomalous transport can be dominant over a large region of the plasma in an optimized stellarator.^10^ On the other hand, at least some types of turbulent transport might be reduced or even absent in W7-X,^40^ and one can of course hope that operational modes with reduced anomalous transport (see, e.g., Ref. 41) will be discovered. The role of anomalous transport in present and next-generation stellarators is a topic of great interest (see, e.g., Ref. 42).

Keeping the potential underestimation of anomalous transport in mind, we will use results from the code in the following to assess triple products for future operation phases.

For OP1.2, it is expected to have 9 MW of ECRH heating, and central densities up to $1.6\times 10^{20}$ m^–3^, so a previously published code prediction with P= 5 MW of ECRH and $n_{e0}=1.5\times 10^{20}$ m^–3^ (Ref. 32) should represent OP1.2 performance conservatively. From those simulations, we find $T_{i0}=2.8$ keV and $\tau_{E}=0.46$ s for the *low iota* configuration, that is, a predicted triple product of $1.9\times 10^{20}$ m^–3 ^keV s.

**FIG. 7.**
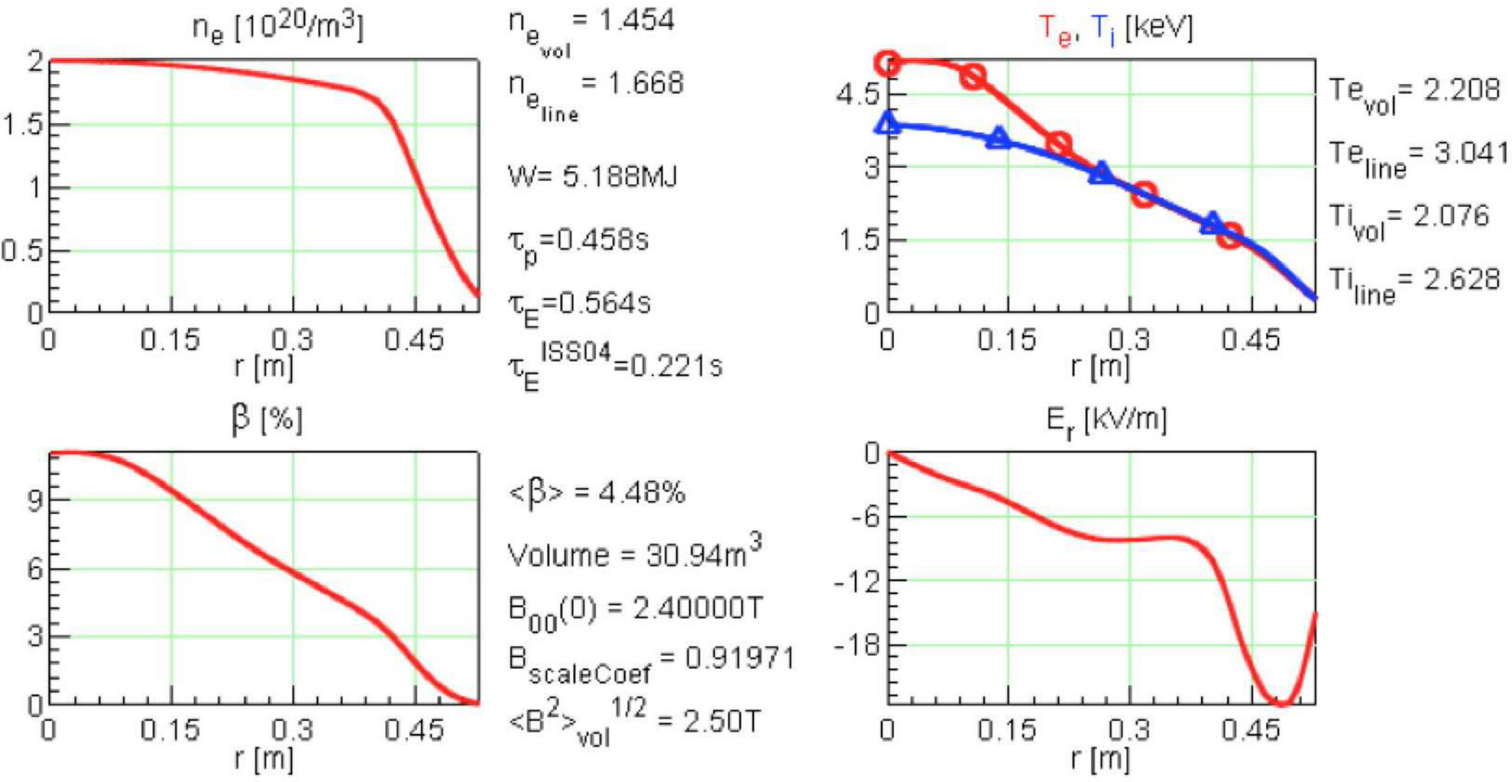
Simulation results from a high density discharge heated with 10 MW of on-axis O2 ECRH are shown here.

In OP2, densities above the X2 heating cutoff at $1.6\times 10^{20}$ m^–3^ will be achieved using O2 heating, and a total heating power up to 20 MW is expected to be available for 10 s pulses, and 10 MW of ECRH for up to 30 min. Simulation results from a high density ($n_{e0}=2.0\times 10^{20}$ m^–3^) discharge heated with 10 MW of ECRH are shown in Figure 7. This is still 20% below the cutoff density for second harmonic ordinary mode (O2) heating. This simulation predicts a triple product of $4.0\times 10^{20}$ m^–3 ^keV s. In the core region, the electron temperature is still noticeably above the ion temperature, despite the high density, but not enough that the CERC feature appears; the plasma is predicted to have a negative radial electric field throughout its volume. The code predicts a substantial improvement over the ISS04 scaling, $\tau_{E}=0.564$ s, compared to $\tau_{E,ISS04}=0.221$ s. This may be taken as a sign of the benefits of the neoclassical optimization, but one should caution again here that the anomalous transport is not fully self-consistently calculated and may be underestimated.

**FIG. 8.**
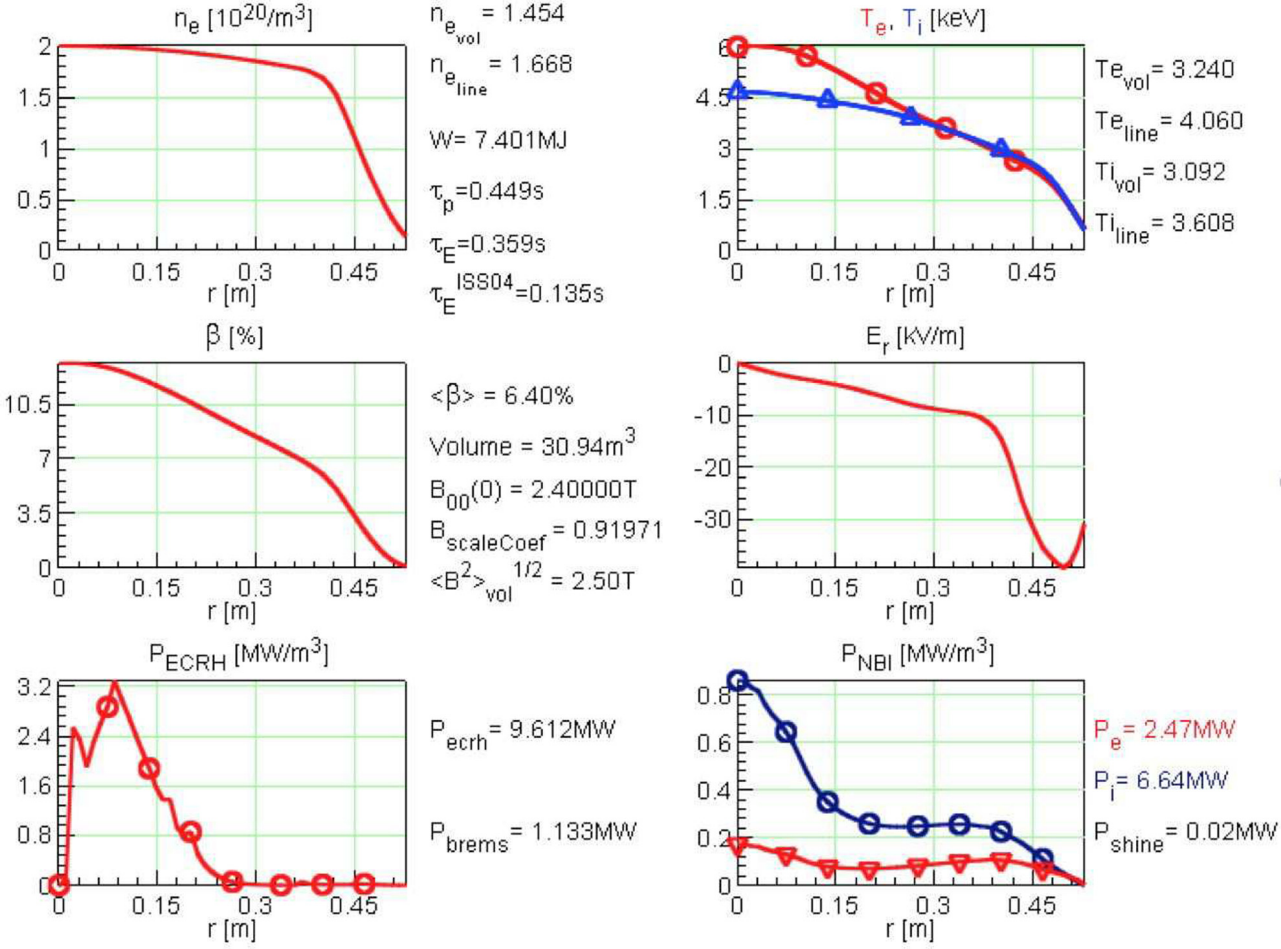
Simulation results from a high density discharge heated with 20 MW is shown, 10 MW of on-axis O2 ECRH and 10 MW of neutral beam heating.

In OP2, it is planned to have up to 10 MW of neutral beam heating in addition to the 10 MW of ECRH. A simulation for such a 20 MW heating scenario is shown in Figure 8. The prediction is likely not directly relevant for operation, since its predicted ⟨β⟩ of 6.4% presumably is not MHD stable,^43^ but if the anomalous transport assumptions are not too optimistic, then this shows that W7-X will be able to test its *β* limits, expected at ⟨β⟩=5% for optimized conditions, with the planned power upgrades to the device. As a side note, this scenario has a predicted triple product of $3.0\times 10^{20}$ m^–3 ^keV s, smaller than the just discussed 10 MW discharge scenario. This is because of the lower confinement time, a result of the deposition profile of the neutral beam heating, which is very broad, and possibly also due to power degradation.

One might get even better triple product results if the O-mode to X-mode to Bernstein-wave (OXB) conversion heating scheme can be realized. This heating scheme, which has been demonstrated in previous stellarators,^44,45^ would allow operation at even higher densities. Also, it is worth pointing out that the achieved and predicted triple product results from W7-X are all for situations with $T_{e0}>T_{i0}$, a condition that will also prevail in a burning D-T fusion plasma since the *α* particles deposit their energy primarily on the electrons. The highest tokamak triple products are achieved in hot ion conditions ($T_{e0}<T_{i0}$).

## COMPARISON TO ALREADY ACHIEVED PARAMETERS ON OTHER DEVICES

**FIG. 9.**
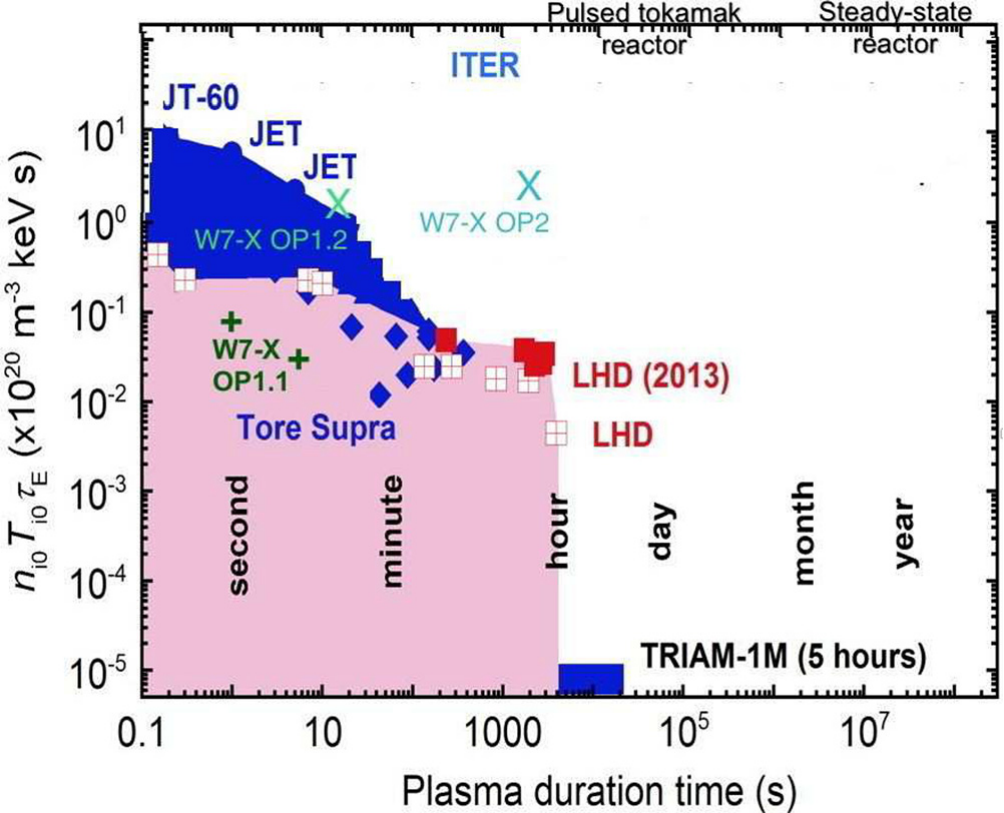
The triple product is plotted versus pulse length in this graph, putting the OP1.1 results in perspective and showing that the combinations of triple product and pulse lengths for OP2 in W7-X will be rather unique. The figure is based on data from Ref. 47 and is an augmented version of one supplied to the authors by T. Morisaki. The pink color indicates regions that LHD can access, and the blue shows the additional parameter space spanned by existing tokamaks.

Figure 9 shows the triple product plotted versus pulse length for a selection of leading devices, and W7-X achieved OP1.1 values (indicated as two dark-green + signs) as well as expected OP1.2 and OP2 values. The expected operating parameters for ITER are also indicated, as are some generic reactor visions. It shows that, in OP2, W7-X will be going beyond what has been achieved to date in fusion experiments, when it comes to the combination of the pulse length and the triple product: the JET and JT-60 tokamaks have achieved significantly higher triple products, but for the triple product predicted for W7-X in OP2, to be held 1800 s, no device to our knowledge has maintained such a triple product for more than 10 s. LHD has achieved pulse lengths of 1800 s and even beyond, but at triple products that are at least a factor of 20 below what is predicted for W7-X for 1800 s discharges. This combination of pulse length and performance manifests itself as technological challenges for the plasma-facing components, the diagnostics, the heating systems, and the control and data acquisition systems.^46^ In these ways, W7-X can also play an important role to help prepare for ITER, which will have similar pulse lengths and similar heat fluxes to the first wall components, but of course many engineering challenges in addition to those that W7-X has.

## SUMMARY

Wendelstein 7-X exceeded the expectations for its first operation phase. Plasma pulses up to 6 s were achieved despite having no divertor. A configurational scan was performed, between two magnetic configurations that differed only slightly in terms of rotational transform ᵼ, but had substantially different effective helical ripple. As expected, they did not show any significant difference in confinement time, since CERC conditions were present, i.e., equilibrium *E *×* B* drift effects healed the orbits of otherwise unconfined ions, an effect seen in other stellarators and in toroidal non-neutral plasmas. First preliminary evidence of the optimisation was indirect—the measured bootstrap current scaled qualitatively as expected as the configurational scan was performed. A triple product of $1\times 10^{19}$ m^–3^ was achieved in OP1.1, and this is expected to increase by an order of magnitude, perhaps as much as a factor of 40, in future operation phases, primarily as a result of the higher density and higher heating power expected.

## References

[1] C. Beidler , G. Grieger , F. Herrnegger , E. Harmeyer , J. Kisslinger , W. Lotz , H. Maassberg , P. Merkel , J. Nührenberg , F. Rau , J. Sapper , F. Sardei , R. Scardovelli , A. Schlüter , and H. Wobig , “ Physics and engineering design for Wendelstein VII-X,” Fusion Sci. Technol. 17, 148–168 (1990).

[2] P. Merkel , Nucl. Fusion 27, 867 (1987). 10.1088/0029-5515/27/5/018

[3] H. Wobig and S. Rehker , A stellarator coil system without helical windings, in *Proceedings of 7th Symposium on Fusion Technology**, Grenoble, France*, October 24–27, 1972, pp. 333–343.

[4] P. Helander , “ Theory of plasma confinement in non-axisymmetric magnetic fields,” Rep. Prog. Phys. 77, 087001 (2014). 10.1088/0034-4885/77/8/087001 25047050

[5] A. H. Boozer , “ Non-axisymmetric magnetic fields and toroidal plasma confinement,” Nucl. Fusion 55, 025001 (2015). 10.1088/0029-5515/55/2/025001

[6] M. Hirsch , J. Baldzuhn , C. Beidler , R. Brakel , R. Burhenn , A. Dinklage , H. Ehmler , M. Endler , V. Erckmann , and Y. Feng , “ Major results from the stellarator Wendelstein 7-AS,” Plasma Phys. Controlled Fusion 50, 053001 (2008). 10.1088/0741-3335/50/5/053001

[7] A. H. Boozer , Phys. Fluids 23, 904 (1980). 10.1063/1.863080

[8] F. S. B. Anderson , A. F. Almagri , D. T. Anderson , P. G. Matthews , J. N. Talmadge , and J. L. Shohet , Fusion Technol. 27, 273 (1995).

[9] S. P. Gerhardt , J. N. Talmadge , J. M. Canik , and D. T. Anderson , Phys. Rev. Lett. 94, 015002 (2005). 10.1103/PhysRevLett.94.015002 15698090

[10] J. M. Canik , D. T. Anderson , F. S. B. Anderson , K. M. Likin , J. N. Talmadge , and K. Zhai , “ Experimental demonstration of improved neoclassical transport with quasihelical symmetry,” Phys. Rev. Lett. 98, 085002 (2007). 10.1103/PhysRevLett.98.085002 17359105

[11] S. Murakami , A. Wakasa , H. Maaßberg , C. D. Beidler , H. Yamada , K. Y. Watanape , and LHD Experimental Group, “ Neoclassical transport optimization of LHD,” Nucl. Fusion 42, L19–L22 (2002). 10.1088/0029-5515/42/11/101

[12] T. Sunn Pedersen , T. Andreeva , H.-S. Bosch , S. Bozhenkov , F. Effenberg , M. Endler , Y. Feng , D. A. Gates , J. Geiger , D. Hartmann , H. Hölbe , M. Jakubowski , R. König , H. P. Laqua , S. Lazerson , M. Otte , M. Preynas , O. Schmitz , T. Stange , Y. Turkin , and the W7-X Team “ Plans for the first plasma operation of Wendelstein 7-X,” Nucl. Fusion 55, 126001 (2015). 10.1088/0029-5515/55/12/126001

[13] S. A. Bozhenkov , M. W. Jakubowski , H. Niemann , S. A. Lazerson , G. A. Wurden , C. Biedermann , G. Kocsis , R. König , F. Pisano , L. Stephey , T. Szepesi , U. Wenzel , T. S. Pedersen , R. C. Wolf , and W7-X Team, “ Symmetrization of W7-X limiter loads with error field correction coils,” Nucl. Fusion (submitted).

[14] A. Peacock , H. Greuner , F. Hurd , J. Kißlinger , R. König , B. Mendelevitch , R. Stadler , F. Schauer , R. Tivey , J. Tretter , C. von Sehren , and M. Ye , “ Progress in the design and development of a test divertor (TDU) for the start of W7-X operation,” Fusion Eng. Des. 84, 1475 (2009). 10.1016/j.fusengdes.2009.01.053

[15] H. Renner , D. Sharma , J. Kißlinger , J. Boscary , H. Grote , and R. Schneider , “ Physical aspects and design of the Wendelstein 7-X Divertor,” Fusion Sci. Technol. 46, 31826 (2004).

[16] T. Wauters , T. Stange , H. P. Laqua , R. Brakel , S. Marsen , D. Moseev , T. Sunn Pedersen , O. Volzke , S. Brezinsek , A. Dinklage , and the W7-X Team, “ Wall Conditioning by ECRH and GDC at the Wendelstein 7-X stellarator,” in *43rd EPS Conference on Plasma Physics, Leuven**, Belgium* (2016), Vol. 40A, p. P4.047.

[17] J. D. Lawson , “ Some criteria for a power producing thermonuclear reactor,” Proc. Phys. Soc. B 70, 6–10 (1957). 10.1088/0370-1301/70/1/303

[18] A. E. Costley , J. Hugill , and P. F. Buxton , “ On the power and size of tokamak fusion pilot plants and reactors,” Nucl. Fusion 55, 033001 (2015). 10.1088/0029-5515/55/3/033001

[19] E. Pasch , M. N. A. Beurskens , S. A. Bozhenkov , G. Fuchert , J. Knauer , and R. C. Wolf , and W7-X Team. “ The Thomson scattering system at Wendelstein 7-X,” Rev. Sci. Instrum. 87, 11E729 (2016). 10.1063/1.4962248 27910540

[20] M. Krychowiak , A. Adnan , A. Alonso , T. Andreeva , J. Baldzuhn , T. Barbui , M. Beurskens , W. Biel , C. Biedermann , B. D. Blackwell *et al.*, “ Overview of diagnostic performance and results for the first operation phase in Wendelstein 7-X (invited),” Rev. Sci. Instrum. 87, 11D304 (2016). 10.1063/1.4964376 27910389

[21] M. R. Stoneking , M. A. Growdon , M. L. Milne , and R. T. Peterson , Phys. Rev. Lett. 92, 095003 (2004). 10.1103/PhysRevLett.92.095003 15089477

[22] J. P. Marler and M. R. Stoneking , Phys. Rev. Lett. 100, 155001 (2008). 10.1103/PhysRevLett.100.155001 18518114

[23] T. Sunn Pedersen and A. H. Boozer , “ Confinement of nonneutral plasmas on magnetic surfaces,” Phys. Rev. Lett. 88, 205002 (2002). 10.1103/PhysRevLett.88.205002 12005572

[24] J. P. Kremer , T. Sunn Pedersen , Q. Marksteiner , and R. G. Lefrancois , “ Experimental confirmation of stable, small debye length pure electron plasma equilibria in a stellarator,” Phys. Rev. Lett. 97, 095003 (2006). 10.1103/PhysRevLett.97.095003 17026372

[25] P. W. Brenner , T. S. Pedersen , X. Sarasola , and M. S. Hahn , Contrib. Plasma Phys. 50, 678 (2010). 10.1002/ctpp.200900003

[26] G. Kuo-Petravic , A. H. Boozer , J. Rome , and R. H. Fowler , “ Numerical evaluation of magnetic coordinates for particle transport studies in asymmetric plasmas,” J. Comput. Phys. 51, 261 (1983). 10.1016/0021-9991(83)90092-X

[27] C. D. Beidler , K. Allmaier , M. Y. Isaev , S. V. Kasilov , W. Kernbichler , G. O. Leitold , H. Maaßberg , D. R. Mikkelsen , S. Murakami , M. Schmidt , D. A. Spong , V. Tribaldos , and A. Wakasa , “ Benchmarking of the mono-energetic transport coefficients—Results from the International Collaboration on Neoclassical Transport in Stellarators (ICNTS),” Nucl. Fusion 51, 076001 (2011). 10.1088/0029-5515/51/7/076001

[28] M. Yokoyama , H. Maaßberg , C. D. Beidler , V. Tribaldos , K. Ida , T. Estrada , F. Castejon , A. Fujisawa , T. Minami , T. Shimozuma , Y. Takeiri , A. Dinklage , S. Murakami , and H. Yamada , “ Core electron-root confinement (CERC) in helical plasmas,” Nucl. Fusion 47, 1213 (2007). 10.1088/0029-5515/47/9/018

[29] M. A. Pedrosa , C. Silva , C. Hidalgo , B. A. Carreras , R. O. Orozco , D. Carralero , and T. J.-I. I. team , “ Evidence of long-distance correlation of fluctuations during edge transitions to improved-confinement regimes in the TJ-II stellarator,” Phys. Rev. Lett. 100, 215003 (2008). 10.1103/PhysRevLett.100.215003 18518613

[30] R. S. Wilcox , B. Ph. van Milligen , C. Hidalgo , D. T. Anderson , J. N. Talmadge , F. S. B. Anderson , and M. Ramisch , “ Measurements of bicoherence and long-range correlations during biasing in the HSX stellarator,” Nucl. Fusion 51, 083048 (2011). 10.1088/0029-5515/51/8/083048

[31] H. Maaßberg , W. Lotz , and J. Nührenberg , Phys. Fluids B 5, 3728 (1993). 10.1063/1.860843

[32] J. Geiger , C. D. Beidler , Y. Feng , H. Maaßberg , N. B. Marushchenko , and Y. Turkin , “ Physics in the magnetic configuration space of W7-X,” Plasma Phys. Controlled Fusion 57, 014004 (2015). 10.1088/0741-3335/57/1/014004

[33] M. Endler , B. Brucker , V. Bykov , A. Cardella , A. Carls , F. Dobmeier , A. Dudek , J. Fellinger , J. Geiger , K. Grosser , O. Grulke , D. Hartmann , D. Hathiramani , K. Höchel , M. Köppen , R. Laube , U. Neuner , X. Peng , K. Rahbarnia , K. Rummel , T. Sieber , S. Thiel , A. Vorkoper , A. Werner , T. Windisch , and M. Y. Ye , “ Engineering design for the magnetic diagnostics of Wendelstein 7-X,” Fusion Eng. Des. 100, 468–494 (2015). 10.1016/j.fusengdes.2015.07.020

[34] H. Yamada , J. H. Harris , A. Dinklage , E. Ascasibar , F. Sano , S. Okamura , J. Talmadge , U. Stroth , A. Kus , S. Murakami *et al.*, Nucl. Fusion 45, 1684 (2005). 10.1088/0029-5515/45/12/024

[35] ITER Physics Basis Editors, “ ITER physics expert groups on confinement and transport and confinement modelling and database,” Nucl. Fusion 39, 2175 (1999). 10.1088/0029-5515/39/12/302

[36] S. Sudo , Y. Takeiri , H. Zushi , F. Sano , K. Itoh , K. Kondo , and A. Iiyoshi , “ Scalings of energy confinement and density limit in stellarator/heliotron devices,” Nucl. Fusion 30, 11 (1990). 10.1088/0029-5515/30/1/002

[37] B. Lipschultz , B. LaBombard , E. S. Marmar , M. M. Pickrell , J. L. Terry , R. Watterson , and S. M. Wolfe , “ MARFES: An edge plasma phenomenon,” Nucl. Fusion 24, 977 (1984). 10.1088/0029-5515/24/8/002

[38] F. Warmer , C. D. Beidler , A. Dinklage , R. Wolf , and the W7-X Team, “ From W7-X to a HELIAS fusion power plant: Motivation and options for an intermediate-step burning-plasma stellarator,” Plasma Phys. Controlled Fusion 58, 074006 (2016). 10.1088/0741-3335/58/7/074006

[39] Y. Turkin , C. D. Beidler , H. Maaßberg , S. Murakami , V. Tribaldos , and A. Wakasa , Phys. Plasmas 18, 022505 (2011). 10.1063/1.3553025

[40] J. H. E. Proll , P. Helander , J. W. Connor , and G. G. Plunk , “ Resilience of quasi-isodynamic stellarators against trapped-particle instabilities,” Phys. Rev. Lett. 108, 245002 (2012). 10.1103/PhysRevLett.108.245002 23004281

[41] F. Wagner , M. Hirsch , H.-J. Hartfuss , H. P. Laqua , and H. Maassberg , “ H-mode and transport barriers in helical systems,” Plasma Phys. Controlled Fusion 48, A217–A239 (2006). 10.1088/0741-3335/48/5A/S21

[42] P. Xanthopoulos , H. E. Mynick , P. Helander , Y. Turkin , G. G. Plunk , F. Jenko , T. Görler , D. Told , T. Bird , and J. H. E. Proll , “ Controlling turbulence in present and future stellarators,” Phys. Rev. Lett. 113, 155001 (2014). 10.1103/PhysRevLett.113.155001 25375712

[43] C. Nührenberg , “ Free-boundary ideal MHD stability of W7-X divertor equilibria,” Nucl. Fusion 56, 076010 (2016). 10.1088/0029-5515/56/7/076010

[44] H. P. Laqua , V. Erckmann , H. J. Hartfu , H. Laqua , and W.-A. S. Team, ECRH Group, “ Resonant and nonresonant electron cyclotron heating at densities above the plasma cutoff by O-X-B mode conversion at the W7-AS stellarator,” Phys. Rev. Lett. 78, 3467 (1997). 10.1103/PhysRevLett.78.3467

[45] M. Otte , D. Andruczyk , E. Holzhauer , J. Howard , R. König , L. Krupnik , H. P. Laqua , O. Lischtschenko , S. Marsen , J. Schacht , J. Urban , Y. Y. Podoba , J. Preinhalter , F. Wagner , G. B. Warr , and A. Zhezhera , “ The WEGA stellarator: Results and prospects,” AIP Conf. Proc. 993, 3 (2008). 10.1063/1.2909160

[46] H.-S. Bosch , R. C. Wolf , T. Andreeva , J. Baldzuhn , D. Birus , T. Bluhm , T. Bräuer , H. Braune , V. Bykov , A. Cardella *et al.*, “ Technical challenges in the construction of the steady-state stellarator Wendelstein 7-X,” Nucl. Fusion 53, 126001 (2013). 10.1088/0029-5515/53/12/126001

[47] Fig. 3.16 and Appendix of Chapter 3, in M. Kikuchi and M. Azumi , *Frontiers in Fusion Research II (Introduction to Modern Tokamak Physics)* ( Springer, Verlag, 2015), ISBN 978-3-319-18905-5.

